# Automated continuous monitoring of inorganic and total mercury in wastewater and other waters by flow-injection analysis and cold-vapour atomic absorption spectrometry

**DOI:** 10.1155/S1463924688000264

**Published:** 1988

**Authors:** S. E. Birnie

**Affiliations:** Associated Octel Company Ltd Research & Development Department South Wirral Ellesmere Port L65 4HF UK

## Abstract

An automated continuous monitoring system for the determination of inorganic and total mercury by flow-injection analysis followed by cold-vapour atomic absorption spectrometry is described. The method uses a typical flow-injection manifold where digestion and reduction of the injected sample takes place. Mercury is removed by aeration from the flowing stream in a specially designed air-liquid separator and swept into a silica cell for absorption measurement at a wavelength of 253.7 nm. A calibration curve up to 10 μg Hg ml^-1^ using three different path length cells is obtained with a detection limit of 0.02 μg Hg ml^-1^. The sampling rate of an injection every 3 min produces 20 results per hour from a flowing stream.


Journal of Automatic Chemistry, Vol. 10, No. 3 (July–September 1988), pp. 140–143

### Automated continuous monitoring of inorganic and total mercury in wastewater and other waters by flow-injection analysis and cold-vapour atomic absorption spectrometry

**S. E. Birnie**

*Associated Octel Company Ltd, Research & Development Department, Ellesmere Port, South Wirral L65 4HF, UK*

*An automated continuous monitoring system for the determination of inorganic and total mercury by flow-injection analysis followed by cold-vapour atomic absorption spectrometry is described. The method uses a typical flow-injection manifold where digestion and reduction of the injected sample takes place. Mercury is removed by aeration from the flowing stream in a specially designed air-liquid separator and swept into a silica cell for absorption measurement at a wavelength of 253·7 nm. A calibration curve up to 10 μg Hg ml$^{-1}$ using three different path length cells is obtained with a detection limit of 0·02 μg Hg ml$^{-1}$. The sampling rate of an injection every 3 min produces 20 results per hour from a flowing stream.*

### Introduction

The determination of mercury in aqueous solution at low concentrations is usually carried out by the Cold Vapour Atomic Absorption (CVAA) method which was introduced by Puleukov *et al.* [1] and Hatch and Ott [2]. The method is based on the chemical reduction of mercury ions to elemental mercury, usually with $SnCl_2$ or $NaBH_4$. The elemental mercury is then swept out of solution with a carrier gas into an absorption cell where the atomic absorption at 253·7 nm is measured. This procedure normally uses a discrete sample method in which all the mercury from a given volume of solution is swept through the absorption cell to produce a peak response. Many improvements to the method, Hawley and Ingle [3], Christmann and Ingle [4] have reduced the detection limit to 2 pg in a 1 ml sample. This method is widely accepted as a standard for determining traces of mercury in aqueous solutions and is well documented.

Relatively few automated procedures for the determination of mercury are described in the literature. Most of these are based on the use of a continuous air-segmented flow system such as those described by El-Awady *et al.* [5], Agemian and Chau [6] and Agemian and DaSilva [7]. Other automated systems described are continuous sample flow by Oda and Ingle [8] and a continuous monitoring system by Goto, Shibakaw, Arita and Ishii [9]. A flow-injection analysis (FIA) method described by DeAndrade, Pasquini, Baccan and Vantoon [10] uses a Teflon membrane phase separator, a detection limit of 0·66 ng and a linear calibration up to 70 ng in a 1 ml sample are obtained with CVAA detector. The CVAA method was initially used in conjunction with FIA by Ruzicka and Hansen [11 and 12] but more recently by Astrom [13] for the determination of bismuth using an atomic absorption/hydride generation system. The present paper describes an automated monitoring method, which allows economic and completely continuous determination of inorganic and total mercuy in wastewater and other waters.

### Experimental

*Apparatus*

A schematic diagram of the flow-injection system is shown in figure 1. The sample, the reagents and the carrier solutions were pumped by a Watson Marlow 202F peristaltic pump using Technicon Tygon pump tubes to maintain the correct flow rate in each feedline. PTFE tubing (0·7 mm i.d.) was used as transmission tubing. Mixing coils were also of PTFE, the first coil (250 cm × 1·5 mm i.d.) was heated to 65 °C in a water bath to ensure complete conversion of any organic mercury to the inorganic state. The second coil (60 cm × 0·7 mm i.d.) was used to mix the reducing solution with the sample stream. Automatic sample injection was accomplished by the use of a timing control switch which activated a pair of Rheodyne solenoid valves (type 7163), which in turn

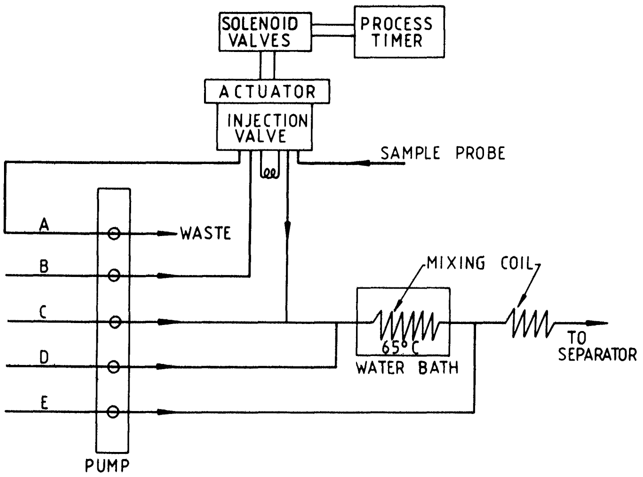

*Figure 1. Configuration of the flow injection analysis system. A: Sample stream, B: Carrier solution, C: Acid reagent, D: Oxidizing reagent, E: Reducing reagent.*

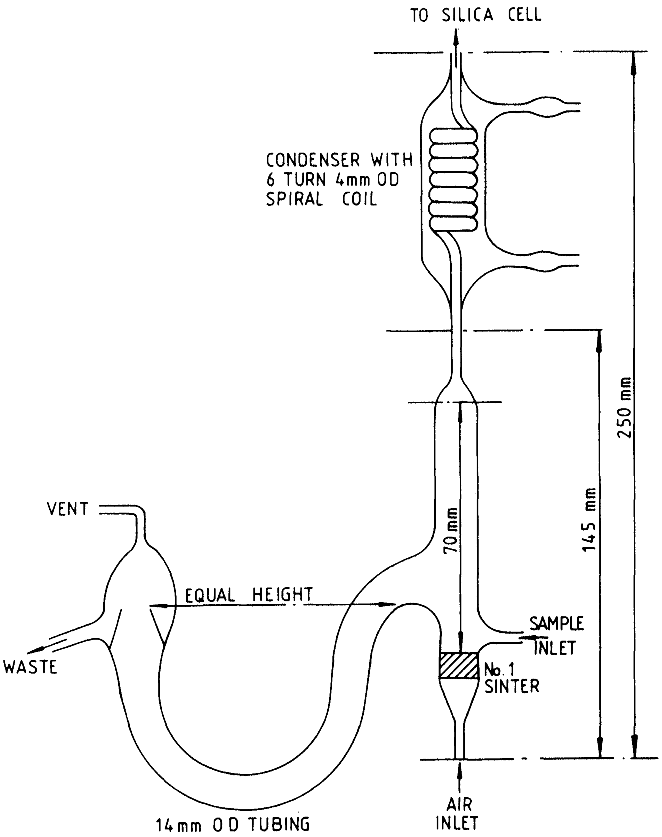

*Figure 2. The air/liquid separator.*

operated a Rheodyne pneumatic activator (type 5001). This activator charged or discharged a Rheodyne rotary sample injection valve (type 5020).

The following sample stream enters a specially designed glass air/liquid separator, shown in figure 2, where, after sample reduction, the mercury vapour was extracted by aeration. The liquid phase was drained to waste by overflowing a U-bend whose dimensions were such that complete extraction of mercury took place and all the gas phase passed through a water cooled condenser into a silica flow cell.

A Perkin-Elmer model 101 atomic absorption spectrophotometer was used to measure the mercury absorption at 253·7 nm using a hollow-cathode mercury lamp source. The signal from this was recorded as sharp peaks by a Servoscribe model RE571 recorder.

*Reagents*

All reagents used were of analytical grade, unless otherwise stated. Distilled water was used for all solution preparations. The reagent solution and their concentrations are as follows:

(1) Carrier reagent, 1% v/v nitric acid solution.

(2) Acid reagent, 50% v/v sulphuric acid solution.

(3) Oxidizing reagent, 4% m/v potassium persulphate solution, laboratory reagent, low in mercury grade (BDH Ltd).

(4) Reducing reagent, 10% m/v tin (11) chloride dissolved in 10% v/v hydrochloric acid solution, Spectrosol low in mercury grade (BDH Ltd).

Mercury standard solutions in the range 2–10 μg Hg $ml^{-1}$ were prepared by suitable dilution of 1 mg Hg $ml^{-1}$ Spectrosol grade solution (BDH Ltd). All the dilute mercury standards were prepared in a matrix similar to the solution to be analysed.

*Procedure*

A flow diagram of the manifold for continuous mercury monitoring is given in figure 1. The procedure for the determination of *inorganic mercury* is as follows.

The sample stream and the carrier reagent are continuously pumped at a flow rate of 1·0 and 2·9 ml $min^{-1}$, respectively. After injection of the sample, the stream is mixed first with the acid reagent and then with water at a flow rate of 0·6 ml $min^{-1}$ for each. The stream is passed into the reaction coil for further mixing. The sample stream is mixed with the reducing reagent at a flow rate of 0·8 ml $min^{-1}$ and passes into the second reaction coil. Here the mercury (11) in the sample is reduced to the elemental state. The mixed stream is introduced into the specially designed air-liquid separator where it is volatilized by aeration at a flow rate of 50 ml $min^{-1}$. The surplus liquid is allowed to drain to waste and all the vapour is carried by the air through the cold water cooled condenser which removes excess water vapour before passage of the vapour to the silica flow cell placed in the atomic absorption spectrophotometer operated at wavelength of 253·7 nm. The absorbance of the mercury is continuously recorded for each sample injection to determine the inorganic mercury present.

The procedure for the determination of *total mercury* is as follows.

The sample stream and the carrier reagent are continuously pumped at a flow rate of 1·0 and 2·9 ml $min^{-1}$ respectively. After injection of the sample, the stream is mixed first with the acid reagent and then with the oxidizing reagent at a flow rate of 0·6 ml $min^{-1}$ for each. The stream is passed into the reaction coil, placed in a water bath at 65 °C for sample digestion. In the reaction coil, organic compounds present in the sample are decomposed and all the organic mercury is converted to the inorganic state. The digested sample stream is mixed with the reducing reagent at a flow rate of 0·8 ml $min^{-1}$ and then passes into the second reaction coil. The absorbance of the mercury is measured in the manner described above for the determination of inorganic mercury.

### Results and discussion

The air-liquid separator shown in figure 2 was designed so that maximum aeration of the sample stream occurs as it flows through the separator. This was achieved by allowing the sample stream to have sufficient residence time to completely vaporize all the mercury present. The overflow after the aeration sinter was of equal height to

| CELL DIMENSION (mm) | MERCURY RANGE (µg Hg ml$^{-1}$) |
|---|---|
| 30 x 25 o.d. | 0 to 10 |
| 95 x 25 o.d. | 0 to 3 |
| 150 x 15 o.d. | 0 to 1 |

*Figure 3. Effect of varying the dimensions of the flow cell.*

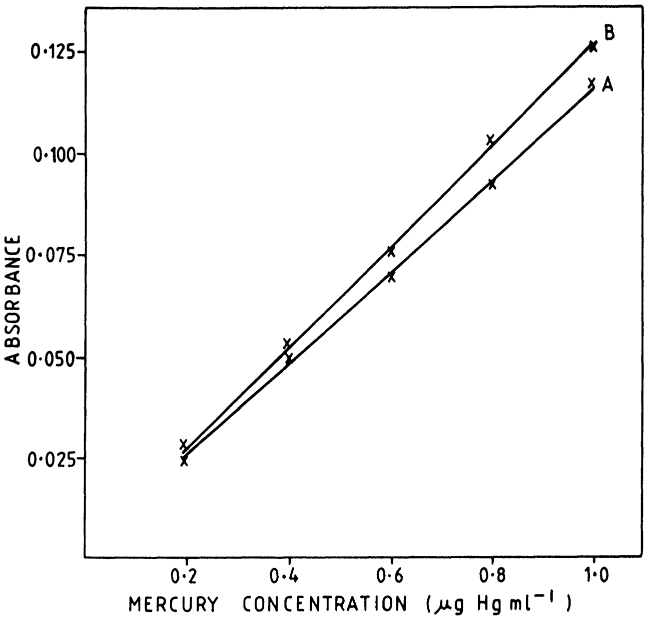

*Figure 4. Comparison of inorganic and methyl mercury absorbance signal response: A = inorganic mercury, B = methyl mercury.*

the overflow from the waste, ensuring that no back pressure was produced which would cause uneven generation of mercury vapour.

Figure 3 shows the range of the method for varying cell dimensions. The flow cell must be constructed with silica glass end windows for operation at a wavelength of 253·7 nm. Mercury concentrations from 0 to 10 µg Hg ml$^{-1}$ can be determined directly without pre-dilution using three different flow cells.

Figure 4 shows a comparison of absorbance signal responses to inorganic and methyl mercury using the total mercury method. A similar response is obtained up to 0·4 µg Hg ml$^{-1}$ but between 0·6 and 1·0 µg Hg ml$^{-1}$ a difference of up to 15% is observed. For a total mercury determination in samples such as wastewater from an industrial process generally the major mercury species is inorganic mercury so it is satisfactory to use inorganic mercury solutions for calibration purposes.

Figure 5 shows typical signal profiles using the optimized conditions. The linear equation for a calibration curve up to 1·0 µg Hg ml$^{-1}$ was $Y$ (absorbance units) = 0·228$x$ +

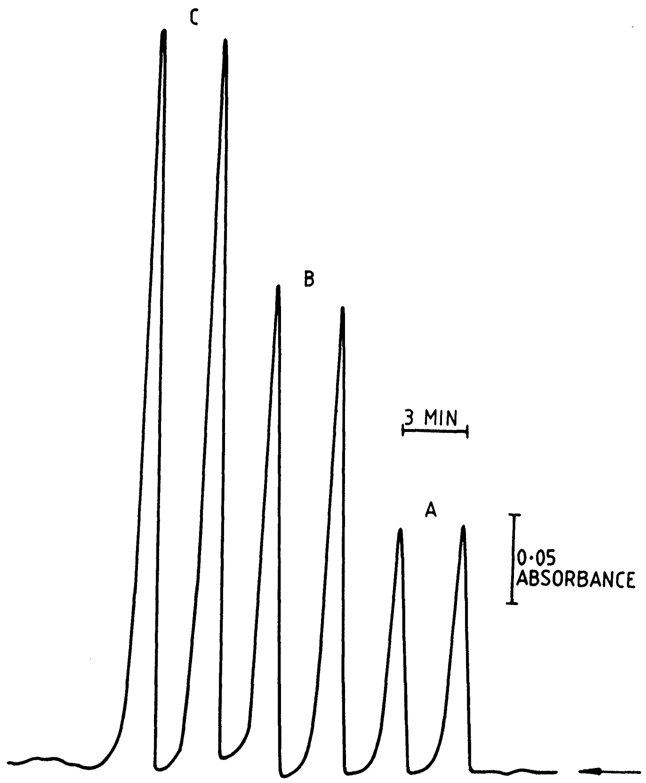

*Figure 5. Typical flow injection signal profiles, A = 2 µg Hg ml$^{-1}$, B = 4 µg Hg ml$^{-1}$, C = 6 µg Hg ml$^{-1}$.*

0·005 where $x$ is the mercury concentration in µg ml$^{-1}$ and has a correlation coefficient of 0·998. The limit of detection (defined as two times the average of the base-line signal) was 0·02 µg Hg ml$^{-1}$. The relative standard deviation, estimated from 30 replicate determinations was 0·93% absorbance units calculated at a level of 6 µg ml$^{-1}$ of mercury.

Some elements which may occur in natural and wastewaters can cause interference in the procedure described which could result in a depression of the mercury FIA signal. Sulphide may combine with the mercury as HgS and not be reduced to the elemental state. Chloride is oxidized by persulphate to liberate chlorine gas which will reduce the signal. Hypochlorite has the same effect but this can be eliminated by boiling with potassium bromide prior to injection. Selenium also depresses the signal, probably due to the formation of mercury selenide, and copper ions have the same effect. The noble metals (palladium, platinum, gold and silver) form amalgams with mercury.

### Conclusion

The proposed method is suitable for the continuous monitoring of inorganic or total soluble mercury in wastewater and other waters. The determination could be made at a sampling frequency of 20 samples per hour. The amount of reagents required are much less than those previously reported in conventional auto-analyser methods.

### Acknowledgements

The author wishes to thank his colleagues, Mr G. Rowlands and Mr P. Scarisbrick, for their co-operation during long-term monitoring trials. Also Mr D. Young for check analysis carried out during development of the method, and Mr F. Wainwright for construction of the air-liquid separator and flow cells, and, finally, the Associated Octel Company for permission to publish this paper.

### References

1. Poluekov, N. S., Vitkum, R. A. and Zelyukova Yu. V., *Zh. Anal. Khim*, **19** (1964), 937.
2. Hatch, R. and Ott, W. L., *Analytical Chemistry*, **40** (1968), 2085.
3. Hawley, J. E. and Ingle, J. D., *Analytical Chemistry*, **46** (1974), 719.
4. Christmann, D. R. and Ingle, J. D., *Analytica Chimica Acta*, **86** (1976), 53.
5. El-Awady, A. A., Miller, R. B. and Carter, M. J., *Analytical Chemistry*, **48** (1976), 110.
6. Agemian, H. and Chau, A. S. Y., *Analytical Chemistry*, **50** (1978), 13.
7. Agamian, H. and DaSilva, J. A., *Analytical Chimica Acta*, **104** (1979), 285.
8. Oda, C. E. and Ingle, J. D., *Analytical Chemistry*, **53** (1981), 2030.
9. Goto, M., Shibakawa, T., Arita, T. and Ishii, D., *Analytica Chimica Acta*, **140** (1982), 179.
10. DeAndrade, J. C., Pasquini, C., Baccan, N. and VanLoon, J. C., *Spectrochimica Acta*, **38B** (10), (1983), 1329.
11. Ruzicka, J. and Hansen, E. H., *Analytica Chimica Acta*, **78** (1975), 145.
12. Ruzicka, J. and Hansen, E. H., *Flow Injection Analysis* (Wiley, New York, 1981).
13. Astrom, O., *Analytical Chemistry*, **54** (1982), 190.

### THIRD INTERNATIONAL SYMPOSIUM ON KINETICS IN ANALYTICAL CHEMISTRY

*To be held in Dubrovnik, Yugoslavia, from 25 to 28 September 1989*

The International Symposium on Kinetics in Analytical Chemistry 1989 ( KAC'89 ) will be held in Cavtat (Dubrovnik) from 25 September to 28 September 1989. The Symposium is the next in the series of triennial conferences, initiated in Cordoba in Spain in 1983, as a result of rapid development in kinetic methods of analysis. The success of the first conference prompted the second meeting which was held in Preveza, Greece in 1986.

*Scientific programme*

The scientific programme will be organized around plenary, invited and contributed papers and posters. The scope of the symposium will be similar to that of the earlier ones, and will include catalytic (enzymatic or non-enzymatic) and non-catalytic methods, differential reaction rate methods, unsegmented flow methods, and any other kinetic aspect of analytical interest.

*The Scientific Committee includes:*

Professor H. A. Mottola (Stillwater, Oklahoma, USA)
Professor G. Werner (Leipzig, GDR)
Professor M. Valcárcel (Córdoba, Spain)
Professor M. I. Karayannis (loannina, Greece)
Professor G. A. Milovanović (Belgrade, Yugoslavia)
Professor F. F. Gaál (Novi Sad, Yugoslavia)

*Language*

The offical language of the Symposium will be English.

*General information and social programme*

The Symposium will be held in the Croatia Hotel in Cavtat, a small town on the Adriatic, close to Dubrovnik. A varied social programme is being prepared to accompany KAC'89 and will include a Welcome Party, a Symposium Banquet and several tours. Post-Symposium excursions will be also arranged.

*Travel Agency*

The official travel agency for KAC'89 (accomodation and post-symposium tours) will be YUGOTOURS, Congress Department Dure Dakovića 31, 11 000 Belgrade, Yugoslavia.

*Further information from Professor Gordana A. Milovanović, Department of Chemistry, University of Belgrade, KAC, P. O. Box 550, 11001 Belgrade, Yugoslavia.*

